# Mini OPCAB in severe COPD—world’s first report of three cases

**DOI:** 10.1093/jscr/rjag907

**Published:** 2026-10-12

**Authors:** Ganesh Kumar K Ammannaya, Federico Jose Benetti

**Affiliations:** Department of Cardiovascular & Thoracic Surgery, Bhavnagar Institute of Medical Sciences Hospital, Bhavnagar, Gujarat 364001, India; Founder & Director, Benetti Foundation, Rosario, Argentina

**Keywords:** mini OPCAB, COPD, OPCAB, minimally invasive CABG, ERAS

## Abstract

Chronic obstructive pulmonary disease (COPD) among patients requiring coronary artery bypass graft is common and is associated with worse outcomes. Although off pump coronary artery bypass (OPCAB) ameliorates cardiopulmonary bypass related derangements, COPD is still an independent risk factor for adverse outcomes. As minimally invasive direct coronary artery bypass is an absolute contraindication in COPD, an alternative minimally invasive approach is imperative to optimize outcomes. We present the world’s first reported cases in the literature of Mini OPCAB performed successfully in three patients with severe COPD that received left internal mammary artery to left anterior descending artery (LAD) revascularization. All three patients were discharged on the first post-operative day with nil complications. All three patients were well at 3 months follow-up, having returned to their normal daily routine by 3 weeks with normal wound healing and without hospital readmission. Therefore, Mini OPCAB as a minimally invasive approach seems an attractive and feasible option in severe COPD, particularly those requiring revascularization to single vessel (LAD). This is the first documented report of Mini OPCAB performed successfully in patients with severe COPD.

## Introduction

The global burden of chronic obstructive pulmonary disease (COPD) is 212.3 million cases with an estimated burden of 55 million cases in India making it the second leading cause of deaths in India next only to coronary artery disease [1]. The prevalence of COPD among coronary artery bypass graft (CABG patients varies from 11% to 25.8% [2]. COPD is an independent risk factor for developing postoperative pneumonia, respiratory failure, stroke, renal failure, wound infection, and mortality after CABG [3]. Further, those with obstructive sleep apnea (OSA)–COPD are at higher risks of non-routine discharge, acute kidney injury (AKI), and respiratory failure/prolonged mechanical ventilation [4]. Off pump approach is better suited for severe COPD by obviating cardiopulmonary bypass (CPB)–related morbidity. Minimally invasive direct coronary artery bypass (MIDCAB) is best avoided in patients with severe COPD [5]. Furthermore, severe COPD is considered as an absolute contraindication for MIDCAB by several authors as it precludes single lung ventilation [6]. Therefore, we explore the feasibility of mini off pump coronary artery bypass (OPCAB) approach in patients with severe COPD as a potential minimally invasive approach of choice in the future. An extensive literature search of all major scientific databases including PubMed, Scopus, Web of science, and Embase with all suitable key search words shows that this operation has not been documented till date in severe COPD patients.

## Case report

Here, we present three cases with severe COPD performed with the Mini-OPCAB approach. We used the Global Initiative for Chronic   Obstructive Lung Disease (GOLD) classification to grade COPD severity and excluded the very severe GOLD Grade 4 category in the present series.

**Patient 1:** A 69-year-old man with unstable angina with percutaneous coronary intervention (PCI) to right coronary artery (RCA), left circumflex artery (LCX), with 100% occlusion of left anterior descending artery (LAD) with 55 pack years of smoking history with severe COPD, and AKI (S Creatinine 1.54).

**Patient 2:** A 68-year-old man with history of anterior wall myocardial infarction (MI) with proximal to mid LAD 90% occlusion, not amenable to primary angioplasty with 45 pack years of smoking history with severe COPD with OSA overlap.

**Patient 3:** A 62-year-old man with past history of PCI to RCA (dominant) with unstable angina, with left main 90%, LAD ostial 90% with 48 pack years of smoking history with severe COPD. Diagnosis of COPD was confirmed by a forced expiratory volume in one second (FEV1)/forced vital capacity (FVC) <50% in all three patients. Patient characteristics are detailed in Table 1.

**Table 1:** Patient characteristics.

	Patient 1	Patient 2	Patient 3
**A) Pre-operative characteristics**			
Age (years)	69	68	62
Sex	Male	Male	Male
Ventricular function (EF)	35%	50%	35%
Recent MI	–	AWMI	–
Previous coronary intervention	PCI to RCA, LCX	No	PCI to RCA
Coronary lesions	100% long segment LAD occlusion	Proximal to mid LAD 90% occlusion	Left main 90%, LAD ostial 90%
STS score	4.8	5.4	4.6
Smoking history	55 pack years	45 pack years	48 pack years
Angina (NYHA class)	Class III	Class II	Class II
FEV1/FVC	48% GOLD 3 B	45% GOLD 3 B	49% GOLD 3 A
Comorbid conditions (AKI/DM/HTN/CVA)	AKI (S Creatinine 1.54)	–	Previous AKI
**B) Intra-operative characteristics**			
Conduit	LIMA	LIMA	LIMA
Graft	LIMA—proximal to mid LAD	LIMA—mid to distal LAD	LIMA—proximal to mid LAD
Operative time (mins)	121	96	75
ACT	>350 s	>350 s	>350 s
CPB	Off pump	Off pump	Off pump
Intracoronary shunt	1.50	2.0	1.50
Need for IABP	No	No	No
Complications	–	–	–
**C) Post-operative characteristics**			
Time to extubation (min)	180	155	123
Time to ambulation (h)	15	17	12
Chest tube drainage (ml)	100	200	150
Length of stay (h)	32	30	27
Complications	–	–	–

**Surgical technique:** All three patients received Mini OPCAB through lower mini sternotomy as described by Benetti [7, 8] with a special retractor with 8–10 cm skin incision. Left internal mammary artery (LIMA) was harvested up to a length of 10 cm as shown in Fig. 1.

**Figure 1:**
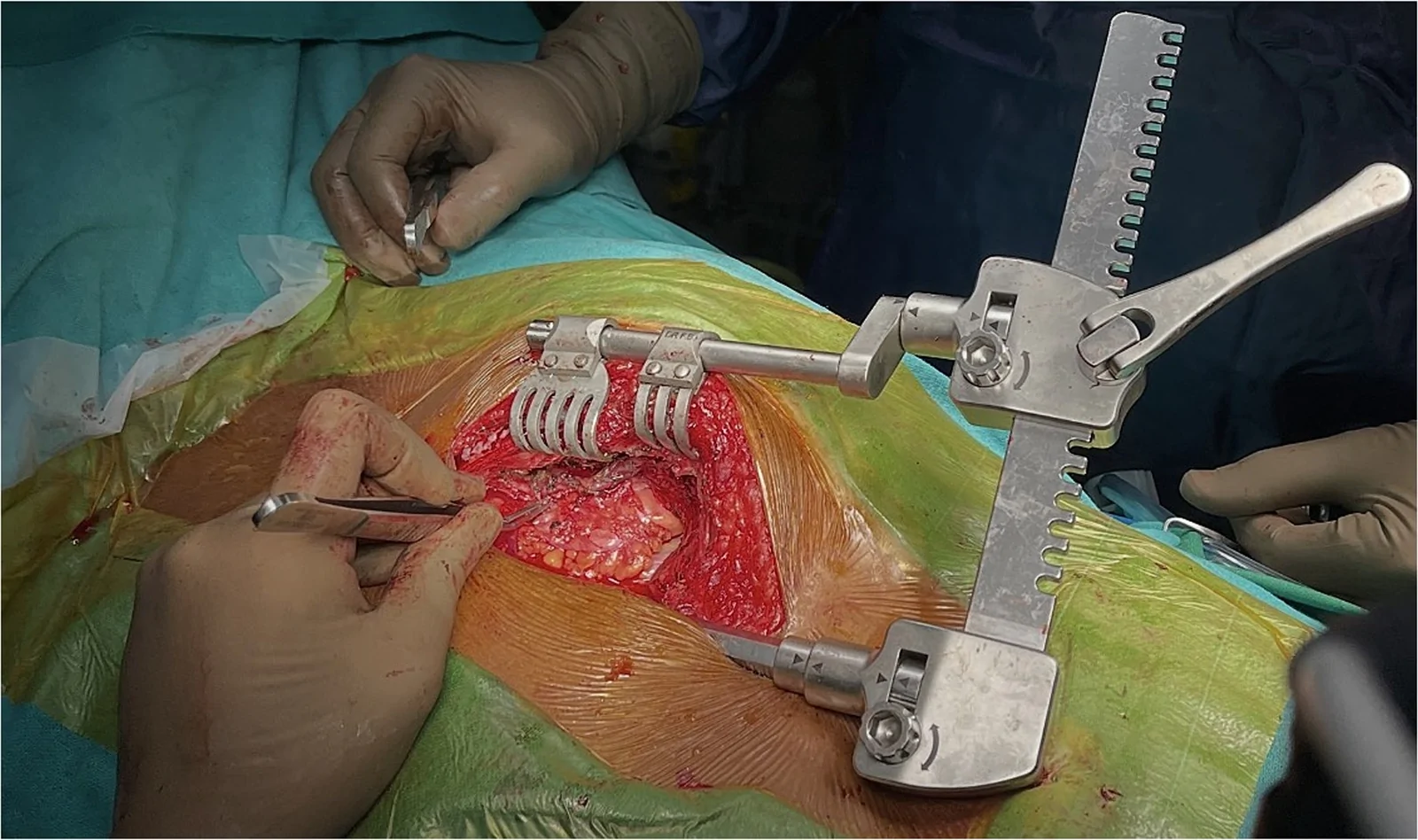
LIMA being harvested with the Mini-OPCAB technique.

Patients 1 and 3 received LIMA-LAD graft to proximal to mid LAD. Patient 2 received LIMA-LAD graft to mid to distal LAD as shown in Fig. 2. Chest was closed with left pleural, and mediastinal drainage tubes with two sternal wires as shown in Fig. 3.

**Figure 2:**
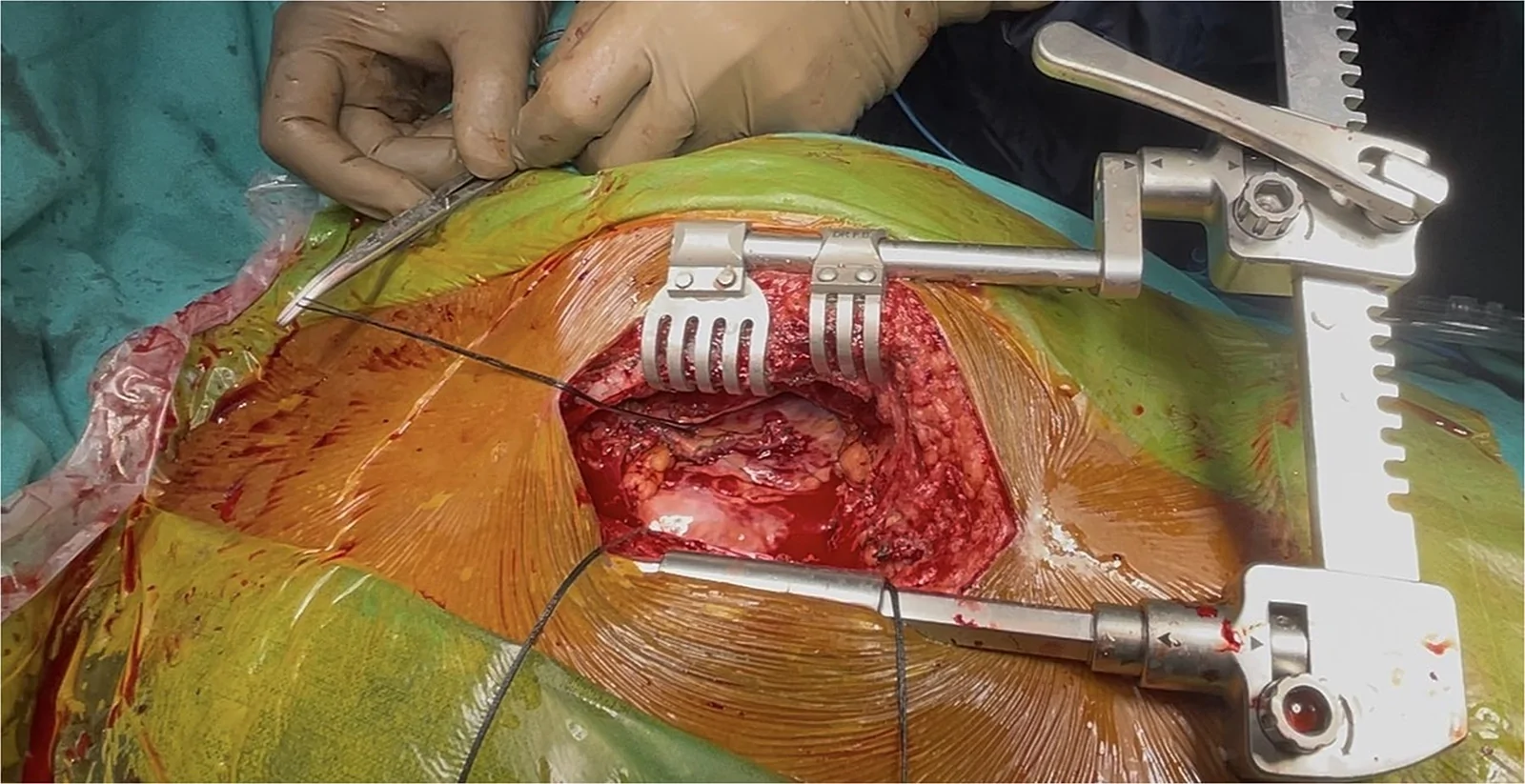
LIMA-mid to distal LAD anastomosis.

**Figure 3:**
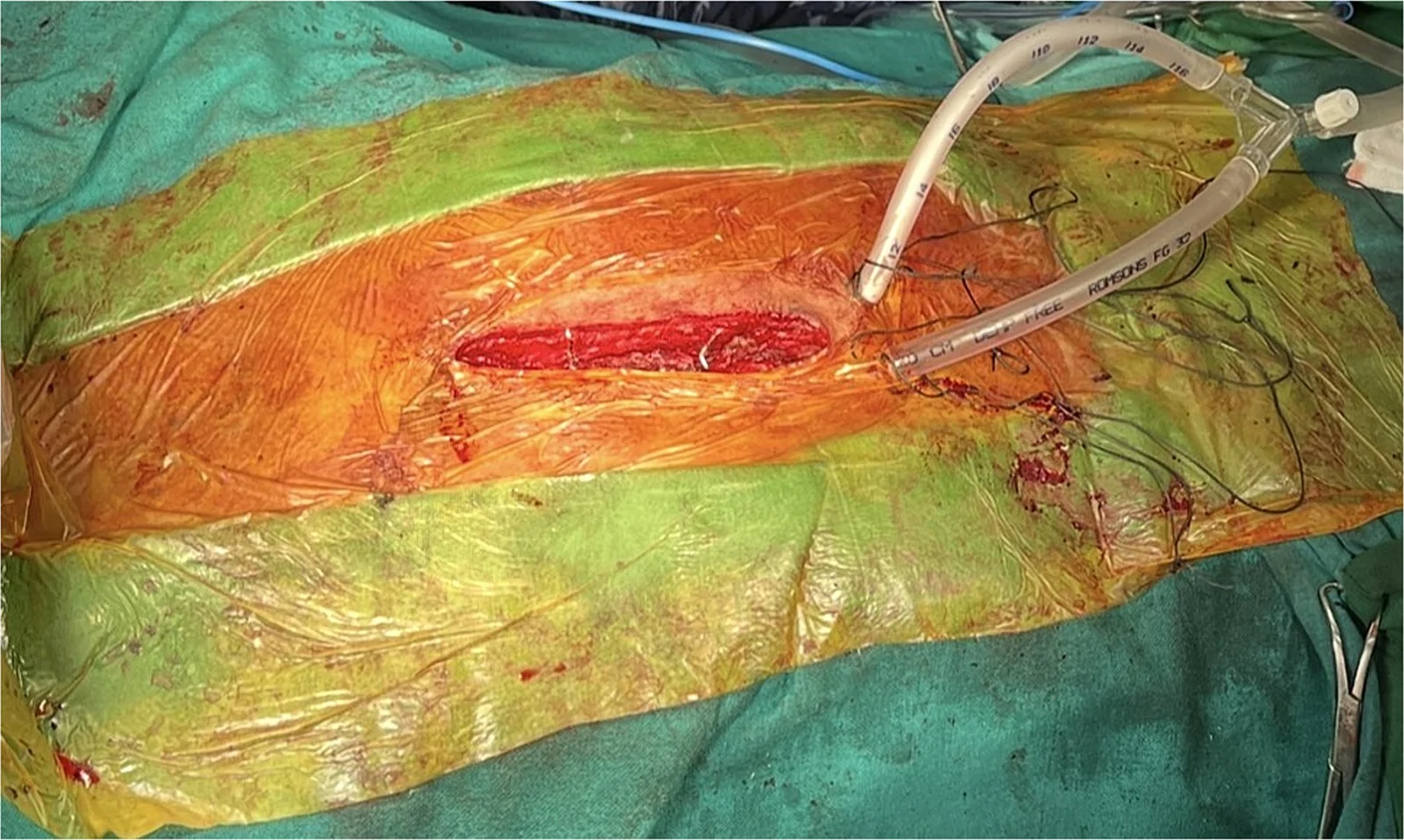
Lower chest closed with two wires.

**Post-operative course:** Patients 1, 2, and 3 were weaned off ventilator and extubated in 180, 155, and 123 min, respectively. All three patients maintained stable hemodynamics with minimal inotropes (<0.01 mic) tapered by the end of post-operative day (POD)-0. Total chest tube drainage in the three patients were 100, 200, and 150 ml, respectively, with all patients having their mediastinal drain removed on the morning of POD-1 and left pleural drain removed after mobilization and chest X-ray later on POD 1. All three patients were discharged by the evening of POD-1 with nil complications during hospital stay.

**Follow-up:** All three patients were asymptomatic, doing well at 3 months follow-up, having returned to their normal daily routine at 3 weeks with normal wound healing, without hospital readmission.

## Discussion

Author 2, Benetti, has the credit of performing world’s first minimally invasive CABG (MIDCAB) in 1995 [9]. In 1999, the first minimally invasive CABG through a lower mini sternotomy (xiphoid approach) was reported by Dullum *et al*. [10], including Benetti. The scope of the xiphoid lower mini-sternotomy approach was further expanded to have double mammary multivessel grafts by Benetti as reported in 2009 [11]. Benetti further documented the largest series of Mini OPCAB and refined the technique, making it more reproducible [7, 8].

OPCAB is the preferred approach for treating severe CAD in COPD to avoid the ill effects of CPB. Surgical trauma can be further limited through a minimally invasive OPCAB approach. However, COPD patients are not ideal candidates for MIDCAB as they cannot tolerate single lung ventilation when required. Furthermore, few authors state COPD as an absolute contraindication for MIDCAB [5, 6]. Therefore, it is imperative to have an alternative minimally invasive approach that circumvents the need for single-lung ventilation, in order to optimize outcomes and achieve enhanced recovery after surgery. Therefore, we used Mini-OPCAB approach as the minimally invasive approach to treat patients requiring revascularization to single vessel (LAD) with severe COPD.

Author 1, Ammannaya, routinely performs OPCAB with a fast-track protocol and discharges with a full sternotomy approach in almost all cases including the more complex ones [12, 13]. Author 2, Benetti, having pioneered Mini OPCAB [7, 8, 10, 11] with the largest documented experience in the same, trained Author 1 for this approach. The application of Mini OPCAB in three COPD patients and the findings have been elaborated in the present report.

We find the Mini OPCAB very feasible with early recovery and discharge as reported in the 3 patients operated.

## Conclusion

To the best of our knowledge, this is the first report of Mini-OPCAB surgery being performed successfully in severe COPD. The present report indicates that Mini OPCAB presents an attractive and very feasible minimally invasive surgical option for coronary revascularization, particularly LIMA-LAD in patients with severe COPD that is associated with quicker recovery, minimal morbidity, and earlier return to work. Hence, we may adopt a cautiously optimistic stance with regards to the application of Mini OPCAB in severe COPD, until larger studies with well-designed randomized control trials and long-term follow-ups confirm these initial findings.
